# Improvement of Corrosion Resistance of Stainless Steel Welded Joint Using a Nanostructured Oxide Layer

**DOI:** 10.3390/nano11040838

**Published:** 2021-03-25

**Authors:** Jun Heo, Sang Yoon Lee, Jaewoo Lee, Akram Alfantazi, Sung Oh Cho

**Affiliations:** 1 Department of Nuclear and Quantum Engineering, Korea Advanced Institute of Science and Technology (KAIST), Daejeon 34141, Korea; heojun@kaist.ac.kr (J.H.); sangyoonlee@kaist.ac.kr (S.Y.L.); jw.lee@kaist.ac.kr (J.L.); 2 Department of Chemical Engineering, Khalifa University of Science and Technology, Abu Dhabi 127788, United Arab Emirates; akram.alfantazi@ku.ac.ae

**Keywords:** corrosion resistance, welded joint, nanoporous oxide layer, anodization

## Abstract

In this study, we fabricated a nanoporous oxide layer by anodization to improve corrosion resistance of type 304 stainless steel (SS) gas tungsten arc weld (GTAW). Subsequent heat treatment was performed to eliminate any existing fluorine in the nanoporous oxide layer. Uniform structures and compositions were analyzed with field emission scanning electron microscope (FESEM) and X-ray diffractometer (XRD) measurements. The corrosion resistance of the treated SS was evaluated by applying a potentiodynamic polarization (PDP) technique and electrochemical impedance spectroscopy (EIS). Surface morphologies of welded SS with and without treatment were examined to compare their corrosion behaviors. All results indicate that corrosion resistance was enhanced, making the treatment process highly promising.

## 1. Introduction

Stainless steel can be classified into different types based on its composition. Type 304 SS is the most widely used austenitic SS, which is primarily composed of Fe, Cr, Ni, and Mn. Non-iron elements are added mostly to improve the corrosion and heat resistance of SS [1,2,3]. In particular, chromium, which constitutes over 11% of type 304 SS in mass, is responsible for the formation of passive films [4,5,6] that provide significant protection from corrosion. Furthermore, in the presence of oxygen, chromium is able to repair these passive films [5,7,8]. Overall, type 304 SS exhibits superb mechanical strength [9,10], high ductility [10], and high corrosion [1,8,11,12] and heat resistance [13], making it highly versatile. Due to this versatility, the SS is used in industries involved in mining, petroleum processing, metal processing, nuclear engineering, underwater construction and additive manufacturing [14,15]. However, all these applications require some degree of welding, diminishing the corrosion resistance of the SS.

Although type 304 SS is generally corrosion resistant, it is occasionally susceptible to localized corrosion in chloride environments; pitting [16,17], intergranular corrosion [18], and stress corrosion cracking (SCC) [17,19] can occur in such conditions. Welding SS makes it even more susceptible to corrosion due to the metallurgical and microstructural changes and the mechanical stress induced by the process [20,21,22]. The microstructural phase of type 304 SS is typically austenite. However, the rapid heating and cooling process involved in welding causes the phase to transition from austenite to delta ferrite as reported in past work [21,23]. Additionally, arc welding, the most common form of welding, causes grain coarsening [24,25] and sensitization [26,27] by precipitating chromium carbides along the grain boundaries at heat affected zones (HAZ). Chromium carbide precipitation leads to chromium depletion, which ultimately reduces the corrosion resistance of the SS. Moreover, SCC can occur from the mechanical stress resulting from welding and any additional processing.

To address these issues, several measures are currently taken. Heat treatment coupled with water quenching is widely used to avoid sensitization and sigma-phase embrittlement [28]. Furthermore, low carbon or stabilized grade alloys can also be used to mitigate sensitization [29,30]. Nonetheless, these procedures require the use of complex equipment at ultra-high temperatures and are expensive to undertake. Recently, a few studies were carried out to enhance the corrosion resistance of SS without utilizing the aforementioned methods. In one study [31], polypyrrole (PPA)-based coating containing polydapamine functionalized carbon powder (C-PDA) was applied, while, in another study [32], graphene oxide (GO) coating was applied. These studies provided great insight into how coating could be used to enhance the corrosion resistance of SS.

In this paper, we propose a simple approach to improving corrosion resistance of type 304 SS weld in a chloride environment using a one-step anodization process. A nanoporous oxide layer was fabricated on the surface of type 304 SS with GTAWs via anodization, after which, the SS was heat-treated to eliminate any existing fluorine. Results show that the anodization process coupled with the heat treatment provides significant corrosion protection to the SS and the welds. Furthermore, the proposed methodology has distinct advantages of being simple, safe, low-cost, and versatile, making it highly promising in numerous fields [33,34,35].

## 2. Materials and Methods

### 2.1. Sample Preparation

**Figure 1:**
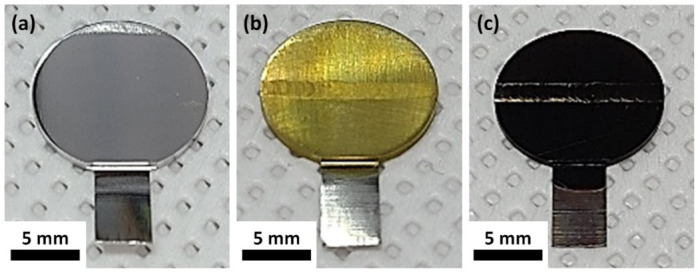
Digital images of welded SS (**a**), anodized welded SS (**b**), and heat-treated anodized welded SS (**c**).

Type 304 SS comprising 18 wt% Cr, 8 wt% Ni, 2 wt% Mn and 72 wt% Fe was used in this research. Autogenous gas tungsten arc welding was performed on the SS to create welds with a chemical composition identical to that of the SS [17]. The SS samples were circular in shape with a diameter of 13 mm and a thickness of 1 mm. A handle was attached to each sample to perform the anodization process as shown in Figure 1a. Prior to anodization, the samples were polished with SiC polishing papers and sonicated in acetone diluted with distilled water for 15 min. The samples were then stored in an oven at 60 °C. Ethylene glycol (REAGENTPLUS, ≥99%) and ammonium fluoride (NH_4_F) were obtained from Sigma-Aldrich (St. Louis, MO, USA) and were used to create the electrolyte for anodization. 

### 2.2. Anodization and Heat Treatment

The anodization process was carried out with two electrodes; one SS weld sample served as the working electrode and a platinum sheet (15 mm × 40 mm × 0.5 mm) served as the counter electrode. Anodization was performed at a constant current density of 100 A/m^2^ in an ethylene glycol-based electrolyte solution containing 0.1 M NH_4_F and 0.1 M H_2_O for 7 min. A cooling bath was used to maintain the temperature of the electrolyte solution at 25 °C. After anodization, the samples were submerged in ethanol for 1 h and dried in a vacuum oven at 50 °C.

To eliminate fluorine, heat treatment was carried out on the anodized samples at 500 °C for 1 h in air. To avoid cracking of the oxide films formed on the welded SS samples, a relatively low heating rate of 2 °C/min. was used. After heat treatment, the samples were gradually cooled to room temperature.

### 2.3. Characterization

Surface morphologies of the samples were examined with a field emission scanning electron microscope (FESEM, Hitachi SU5000, Tokyo, Japan). The chemical composition of the fabricated nanoporous oxide layers was determined with a high-resolution powder X-ray diffractometer (XRD, Rigaku, Japan). Cross-sectional data for the nanoporous oxide layers, such as the chemical composition by depth, was obtained by employing a focused ion beam (FIB) and observing with a SEM (Helios Nanolab 450 F1, FEI, Milpitas, CA, USA). Corrosion properties of the samples were evaluated by applying the potentiodynamic polarization (PDP) technique and electrochemical impedance spectroscopy (EIS) using Reference 600 Potentiostat/Galvanostat (Gamry, Warminster, PA, USA). Corrosion rates and all variables related to corrosion were determined by averaging five values obtained from five separate measurements.

### 2.4. Corrosion Test

A conventional three electrode cell system was adopted. One sample with an exposed area of 0.2 cm^2^ was used as the working electrode, a platinum wire was used as the counter electrode, and a saturated calomel electrode was used as the reference electrode. Firstly, PDP test was performed to compare the corrosion susceptibilities of normal SS welds, anodized welds, and heat-treated anodized welds. Prior to testing, stable open circuit potential (OCP) was achieved and maintained for 1200 s. The potential applied during PDP ranged from −600 to 600 mV with respect to the OCP, and the scan rate was set to 0.333 mV/s.

Electrochemical impedance spectroscopy (EIS) was conducted with a sinusoidal amplitude of 10 mV. The impedance spectra were collected with 5 points per decade over a frequency range of 10^5^ to 10^−2^ Hz. The spectra were analyzed using Echem Analyst software (Gamry). All the corrosion tests were carried out at room temperature in artificial seawater with a chemical composition (Table 1) that is nearly identical to that of real seawater.

## 3. Results and Discussion

### 3.1. Anodized SS Weld

**Figure 2:**
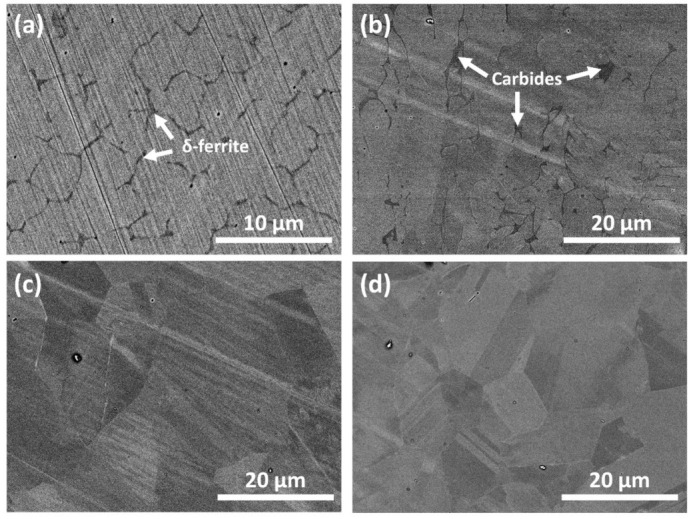
Metallurgical field emission scanning electron microscope (FESEM) images of stainless steel (SS) weld zone showing dendrite structure of δ-ferrite (**a**), chromium carbides (**b**), heat-affected zone (**c**), and base metal (**d**).

**Figure 3:**
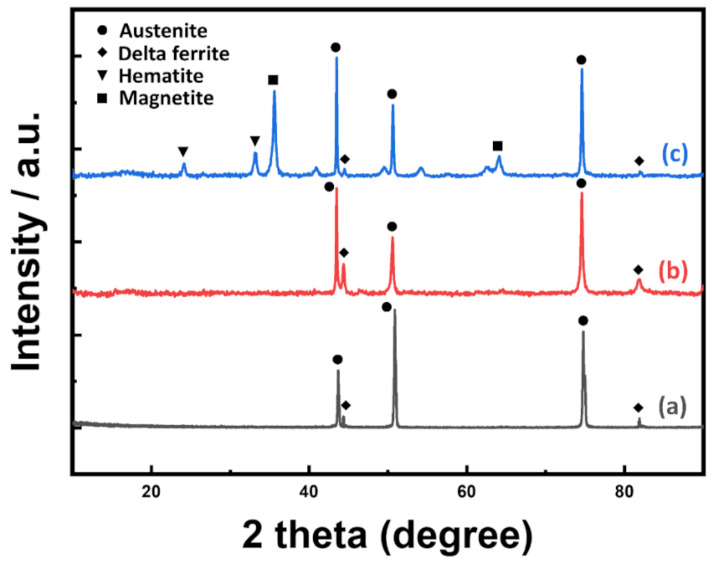
XRD patterns of welded SS (**a**), anodized welded SS (**b**), and heat-treated anodized welded SS (**c**).

A welding joint can have different zones: a weld zone (WZ), a heat affected zone (HAZ), and a base metal (BM) zone. Figure 2 shows the metallographs of the three aforementioned zones. A weld zone can exhibit two phases, austenite and ferrite, as shown in the XRD patterns (Figure 3a). It is widely known that a small amount of δ-ferrite is necessary to avoid thermal cracking during cooling [20,21,36]. However, to enhance corrosion resistance, δ-ferrite content has to be reduced. δ-ferrite traps more chromium than austenite, resulting in insufficient chromium content adjacent to the grain boundaries of the former [37,38]. Moreover, previous works have shown [37,39,40] that chromium carbides (Cr**_23_**C**_6_**) start to precipitate along the δ-ferrite/austenite interfaces at a temperature of 400~800 °C. These phenomena cause chromium-depleted regions to form near grain boundaries, making welded SS susceptible to corrosion. Figure 2a,b are SEM images of a SS weld surface, showing a dendritic structure with dark δ-ferrite encased in a bright austenite matrix and chromium carbide that has precipitated along the δ-ferrite/austenite interfaces, respectively. Figure 2c shows that the grain size in the HAZ is slightly larger than that found in the BM zone. It has been previously reported that the HAZ is very susceptible to pitting corrosion due to the process of recrystallization and stress accumulation resulting from heating [41].

**Figure 4:**
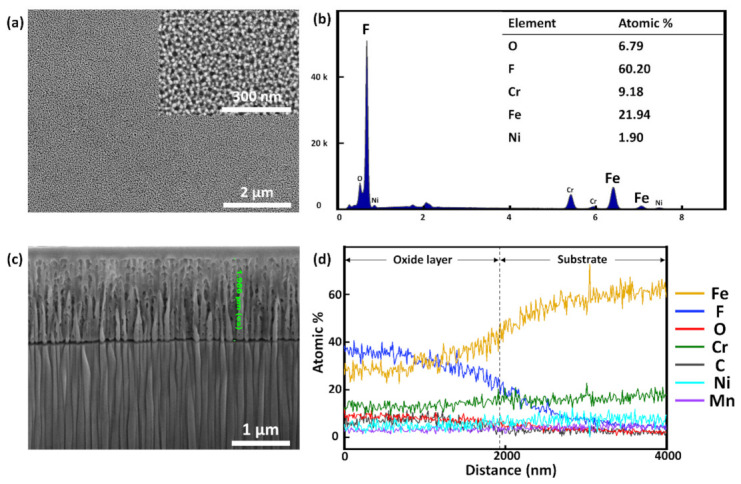
FESEM images showing surface morphologies of anodized welded SS at low and high magnification (**a**) and EDX characterization of the surface (**b**). Cross-sectional FESEM image of anodized welded SS (**c**) and EDX line spectrum of the cross section (**d**).

A SS weld was electrochemically anodized in NH_4_F-based electrolyte for less than 10 min at 298 K and at a constant current density of 100 A/m**^2^**. The current was kept constant to fabricate an oxide layer with uniform nanopores. A distinct color change of the SS surface was observed after anodization (Figure 1b), and FESEM images taken of the surface (Figure 4a) revealed that a nanoporous oxide layer with an average pore diameter of nearly 40 ± 5 nm was fabricated. The pore diameter was determined from averaging values obtained from 50 separate measurements utilizing the built-in software of the FESEM used in this work. The fabricated pores were relatively uniform. EDX measurement (Figure 4b) indicated that the oxide layer was primarily composed of iron and fluorine. The major presence of Fe can be explained by the fact that Fe is the primary constituent of SS. As for F, its presence in the electrolyte can be used to explain its presence in the oxide layer. The equations below show the oxidation and dissolution reactions taking place during anodization.

2H_2_O → O_2_ + 4H^+^ + 4e^−^

2Fe + 3/2O_2_ → Fe_2_O_3_

Fe_2_O_3_ + 6H^+^ + 12F^−^ → 2[FeF_3_]3^−^ + 3H_2_O

During anodization, nanopores were fabricated by dissolution of fluorine, indicating that the competition between oxidation and F dissolution determines the morphology of the resulting nanoporous structure. Cross-sectional FESEM images show that the average length obtained from five measurements of the nanopores was 1.986 ± 0.102 μm (Figure 4c). The oxide layer can be clearly distinguished from the substrate as the former exhibits much higher F and O contents than the latter (Figure 4d).

### 3.2. Heat Treatment

**Figure 5:**
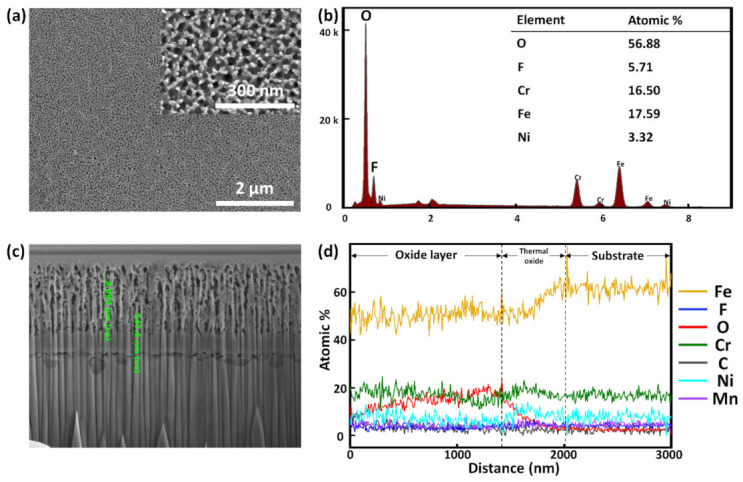
FESEM images showing surface morphologies of heat-treated anodized welded SS at low and high magnification (**a**) and EDX characterization of the surface (**b**). Cross-sectional FESEM image of anodized welded SS (**c**) and EDX line spectrum of the cross section (**d**).

The anodization process creates a nanoporous oxide layer with a high F content. The F compounds found in the oxide layer seriously degrade the layer when it is exposed to an aqueous environment for an extended period. Thus, to eliminate these detrimental compounds, heat treatment was performed at 500 °C for 1 h in air. FESEM images of the heat-treated sample surface (Figure 5a) indicate that the morphology of the nanoporous structure remains relatively unaltered, but the pore diameter increases slightly to about 58 ± 11 nm after heat treatment. The increased pore diameter was determined by averaging values obtained from 50 separate measurements using the built-in software of the FESEM. EDX measurement (Figure 5b) shows that heat treatment causes the F content to decrease to a negligible amount, while steeply increasing the oxygen content. Furthermore, the heat treatment process generated phases [42] other than austenite or δ-ferrite in the nanoporous oxide layer that was largely amorphous prior to the treatment. XRD measurements (Figure 3c) show that the new phases that formed were hematite (Fe**_2_**O**_3_**) and magnetite (Fe**_3_**O**_4_**); of the two, the latter was much more prevalent. Due to its chemical stability [43,44], the magnetite provides significant protection to the fabricated nanoporous oxide layer.

Cross-sectional analysis of the heat-treated sample (Figure 5c,d) showed that the thickness of the overall oxide layer remained relatively unchanged from the thickness prior to the heat treatment, which was approximately 2 μm (Figure 5c). However, the treatment caused a non-porous thermal oxide layer with a thickness of nearly 547.8 ± 6.2 nm to form underneath the nanoporous oxide layer (Figure 5c), slightly thinning the latter layer. The thickness value of the non-porous oxide layer was determined by averaging values obtained from five measurements using the built-in software of the FESEM. Further oxidation will be difficult due to the presence of this compact thermal oxide layer. EDX measurement (Figure 5d) shows that the heat treatment drastically eliminates fluorine, causing further thinning of the nanoporous layer.

### 3.3. Potentiodynamic Polarization Test

Potentiodynamic polarization (PDP) tests were performed on a plain SS weld, an anodized welded SS, and a heat-treated anodized welded SS in artificial seawater. By extrapolating from the fitted anodic and cathodic Tafel plots, corrosion potential (E_corr_), and corrosion current (i_corr_) can be calculated. E_corr_ and i_corr_ quantify the likelihood of corrosion and the severity of incurred corrosion, respectively. Corrosion rate (CR) can be calculated by inputting i_corr_. Pitting potential (E_pit_), also known as breakdown potential, is defined as the potential at which passive film breakdown occurs. E_pit_ can be easily identified in the polarization curve as the starting point of sharp current increase. To accurately determine E_pit_, the international standard (ISO 15158-2014) of setting the potential corresponding to 100 uA/cm**^2^** as E_pit_ was adopted. The interval between E_corr_ and E_pit_ is known as the passive stability region and is the potential range within which stable protective passive film is preserved.

**Figure 6:**
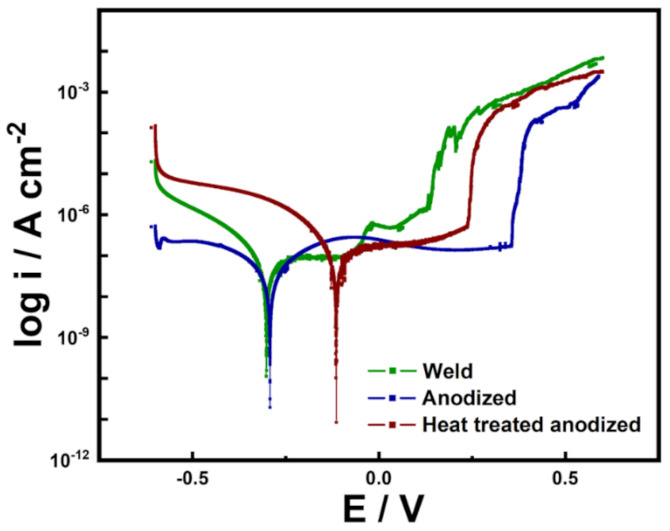
Potentiodynamic polarization curves of welded SS, anodized welded SS, and heat−treated anodized welded SS in artificial seawater medium.

Figure 6 shows the PDP curves of the samples. All relevant parameters are tabulated in Table 2. In comparison to the plain SS weld, the anodized and heat-treated anodized counterparts displayed significantly higher E_corr_, demonstrating that the presence of a nanoporous oxide layer significantly reduces the possibility of corrosion. Among the two samples with high E_corr_, the heat-treated anodized sample was especially high, which seems to be due to the chemically stabilized oxide layer by heat treatment. The i_corr_ values of the plain, the anodized, and the heat-treated anodized welded SS were determined to be 1.788 × 10^−7^ A/cm^2^, 1.118 × 10^−7^ A/cm^2^, and 0.464 × 10^−7^ A/cm^2^, respectively. These values show that the plain SS weld suffers much more severely from corrosion than the other two samples do. The corrosion resistance of the two anodized samples can be attributed to the presence of the protective oxide layer, which hinders the permeation of aggressive ions. The additional corrosion resistance displayed by the heat-treated anodized welded SS can be attributed to the formation of a compact thermal oxide layer and the stabilization of the nanoporous oxide layer resulting from fluorine elimination. Assuming uniform corrosion at the surface, corrosion rate (mm year**^−^**^1^) can be calculated [45] by using the equation below:

(1) $$\mathrm{CR}\left(\mathrm{mm}\;year^{-1}\right)\;=\;\;\frac{i_{corr}\left(Acm^{-2}\right)\times M\left(g\right)}{n\times d\left(gcm^{-3}\right)\times A\left(cm^{2}\right)}\;\times 3272\;$$

As tabulated in Table 2, the corrosion rates of the plain SS weld, the anodized welded SS and the heat-treated anodized welded SS were calculated to be 6.484 × 10^−3^ mmy**^−1^**, 4.055 × 10^−3^ mmy**^−1^** and 1.683 × 10^−3^ mmy**^−1^**, respectively. The anodized samples also exhibited higher E_pit_ values than the plain sample, indicating that the former samples are less likely to experience localized corrosion than the latter. The passive stability region (E_pit_-E_corr_) was also expectedly larger for the anodized samples than the plain sample, demonstrating that anodization enhances passivation behavior [32]. Interestingly, the case of the heat-treated anodized sample showed a lower E_pit_ than that of anodized welded SS, which can be attributed to the fact that a small amount of carbides was generated because the heat treatment temperature slightly spans the carbide formation temperature range. However, the degree was so insignificant that it seems to be negligible. Additionally, even with a large E_pit_, extended submersion in water is not possible with the anodized welded SS; therefore, heat-treated anodized welded SS was evaluated to be the most corrosion resistant sample of the three.

### 3.4. Electrochemical Impedance Spectroscopy

**Figure 7:**
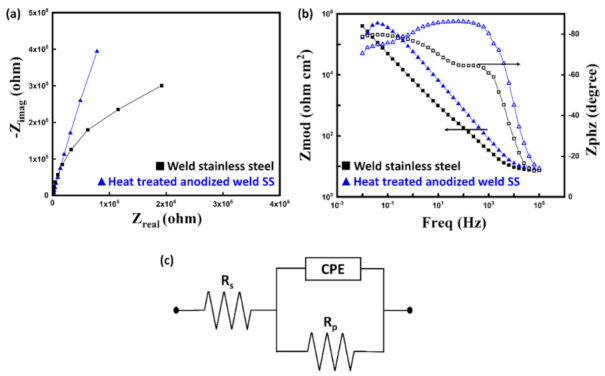
Niquist impedance diagram (**a**), Bode plot (**b**) for welded SS and heat-treated anodized welded SS, and equivalent circuit used for fitting (**c**).

Electrochemical impedance spectroscopy (EIS) is widely used [46,47] to evaluate the corrosion resistance property of anodic films. Figure 7 shows the impedance spectra of the SS weld and the heat-treated anodized welded SS. The linear sections in the Bode plots (Figure 7b) have a slope of near −1 and a phase angle of ~80°. These features are generally observed in metals coated with a passive film [48,49]. At low frequencies, the two plots show small discrepancies, possibly resulting from impurities at the surface [42]. The Niquist plots (Figure 7a) show a larger radius of depressed capacitive semicircle for the heat-treated anodized welded SS than for the plain SS weld, indicating higher impedance of the former. For numerical analysis, a constant phase element (CPE)-based Randles circuit (Figure 7c) was used for fitting [50,51]. The Randles circuit is composed of an active solution resistance (Rs), a polarization resistance (Rp), and CPE. Capacitance was replaced with CPE to obtain a more accurate analysis of corrosion behavior. Impedance of CPE is calculated as follows:

Z_CPE_ = Y**^−^**^1^ (iw)^−n^ (2)

where Y is the admittance magnitude, w is the angular frequency and n signifies the phase shift. The case when n = 0, −1, and 1 describe a pure resistor, a pure inductor and an ideal capacitor, respectively.

All relevant parameters are tabulated in Table 3. The polarization resistances (Rp) of the bare SS weld, and heat-treated anodized welded SS were calculated to be 809 kΩ cm^2^ and 1756 kΩ cm^2^, respectively; a higher polarization resistance (Rp) value indicates superior corrosion resistance. The capacitance can be also calculated using the equation below to evaluate the samples.

C = Y^1/n^ R^(1−n)/n^ (3)

Higher capacitance indicates higher chance of corrosion [52]. The heat-treated anodized sample and the bare sample exhibited capacitance values of 40.54 μF/cm^2^ and 45.04 μF/cm^2^, respectivtabely. As stated before, the fabricated nanoporous oxide layer provides protection from corrosion and the dense thermal oxide layer increases the polarization resistance (Rp) by providing barrier resistance.

### 3.5. Surface Morphology after Corrosion Test

**Figure 8:**
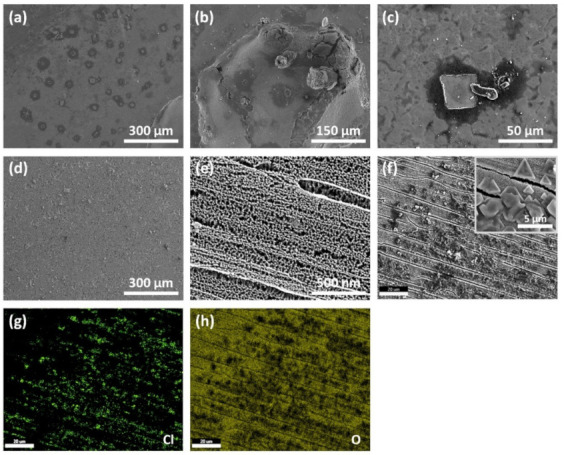
Surface FESEM images of welded SS (**a**–**c**). and of heat-treated anodized welded SS (**d**–**f**) after accelerated corrosion test. EDX characterization of crystals (**f**) on the heat-treated anodized welded SS surface (**g**,**h**).

An electrochemical corrosion test evaluates the performance of samples in a relevant environment by simulating the exposure that the samples would receive if placed in the said environment [53]. Figure 8a–c shows a microscopic image of the SS weld surface after the corrosion test. The images (Figure 8a,c) reveal countless signs of severe corrosion with desorption through the surface shell being observable. Figure 8b shows a large pit, resulting from localized corrosion, found on the surface. Such pitting corrosion can potentially facilitate stress corrosion cracking (SCC) as pits are more susceptible mechanical damage.

Figure 8d–h shows the surface of heat-treated anodized welded SS after the corrosion test. No severe pits are noticeable with only minor pits and signs of corrosion being observable in the low-magnification microscopic image (Figure 8d). Figure 8f shows large amounts of crystals that have accumulated on top of the nanoporous oxide layer, while its inset shows the morphology of the crystals. EDX characterization was performed on the crystals, and the results are shown in the Figure 8g,h; the results indicate that the crystals are chloride-based. The presence of these chloride crystals demonstrates that the stable magnetite (Fe_3_O_4_) layer successfully prevented chlorine ions from penetrating and causing deep corrosion. Figure 8h shows that oxide materials are present at the surface, further cementing the role of the magnetite. Lastly, the structural integrity of the nanopores was maintained after the corrosion (Figure 8e), signifying that the fabricated nanoporous oxide layer is highly durable.

## 4. Conclusions

In this study, corrosion resistance of type 304 SS GTAW joint was enhanced by employing a one-step electrochemical anodization process. The anodization process was conducted in a NH4F-based ethylene glycol electrolyte to produce a nanoporous oxide layer on the surface of a SS weld. To eliminate fluorine from the layer, heat treatment was undergone at 773 K for 1 h. While the heat treatment was successful at removing fluorine, it also induced the oxide layer to crystallize into hematite (Fe_2_O_3_) and magnetite (Fe_3_O_4_) and created a compact non-porous thermal oxide layer underneath the nanoporous layer. Electrochemical corrosion test in artificial seawater showed that the anodization process coupled with the heat treatment significantly enhanced the corrosion resistance of a SS weld. After the corrosion test, the bare SS weld exhibited numerous signs of corrosion products, while the heat-treated anodized welded SS exhibited accumulation of chloride crystals at the surface, signifying that the damaging ions could not penetrate the oxide layer. These results show that the enhancement method described in this paper is highly effective. Furthermore, the anodization method is simple, safe, low-cost and versatile, making it highly promising in many fields.

## Figures and Tables

**Table 1:** Chemical composition of artificial seawater used for corrosion tests.

Element	Composition (g/L)
Cl	19.00
Na	9.72
Mg	1.30
S	0.81
Ca	0.40
K	0.35
Sr	0.007
B	0.004

**Table 2:** Potentiodynamic polarization curve parameters.

Specimen	E_corr_ (mV/SCE)	i_corr_ (10^−7^ A cm^−2^)	E_pit_(mV)	E_pit_ −E_corr_ (mV)	Corrosion Rate (mm year^−1^)
Weld	−301.0 ± 1.7	1.788 ± 0.020	162.6 ± 0.5	463.6 ± 2.2	(6.484 ± 0.073 × 10^−3^
Anodized	−290.0 ± 5.4	1.118 ± 0.102	383.6 ± 4.0	673.6 ± 9.4	(4.055 ± 0.370) × 10^−3^
Heat-treated anodized	−114.0 ± 3.5	0.464 ± 0.094	274.8 ± 3.1	388.8 ± 6.6	(1.683 ± 0.341) × 10^−3^

**Table 3:** Electrochemical impedance spectroscopy (EIS) parameters with Randles circuit coupled with constant phase element (CPE).

	SS Weld	Heat-Treated Anodized Welded SS
R_p_ (kΩ cm^2^)	809	1756
R_s_ (Ω cm^2^)	5.242	5.771
Y (μΩ^−1^s^n^ cm^−2^)	25.62	22.13
n	0.8431	0.8581
C (μF cm^−2^)	45.04	40.54

## Data Availability

Data sharing is not applicable to this article.
